# Triterpene Derivatives as Relevant Scaffold for New Antibiofilm Drugs

**DOI:** 10.3390/biom9020058

**Published:** 2019-02-11

**Authors:** Gloria Narjara Santos da Silva, Muriel Primon-Barros, Alexandre José Macedo, Simone Cristina Baggio Gnoatto

**Affiliations:** 1 Laboratório de Fitoquímica e Síntese Orgânica, Faculdade de Farmácia, Universidade Federal do Rio Grande do Sul, Porto Alegre, Rio Grande do Sul 90610-000, Brazil; narjarags@gmail.com (G.N.S.d.S.); simone.gnoatto@ufrgs.br (S.C.B.G.); 2 Laboratório de Biofilmes e Diversidade, Faculdade de Farmácia and Centro de Biotecnologia, Universidade Federal do Rio Grande do Sul, Porto Alegre, Rio Grande do Sul 91501-970, Brazil; murielprimon@gmail.com

**Keywords:** ursolic acid, betulinic acid, cytotoxicity, pathogenic biofilms, bacterial pathogens

## Abstract

New medicines for the treatment of bacterial biofilm formation are required. For this reason, this study shows the in vitro activity of betulinic acid (BA), ursolic acid (UA) and their twenty derivatives against planktonic and biofilm cells (gram-positive bacterial pathogens: *Enterococcus faecalis*, *Staphylococcus aureus* and *Staphylococcus epidermidis*). We evaluated the antibiofilm activity (through the crystal violet method), as well as the antibacterial activity via absorbance (OD_600_) at concentrations of 5, 25 and 100 µM. Likewise, the cytotoxicity of all compounds was evaluated on a kidney African green monkey (VERO) cell line at the same concentration, by MTT (3-(4,5-Dimethylthiazol-2-yl)-2,5-diphenyltetrazolium bromide) methodology. We verified for the first time whether different groups at carbon 3 (C-3) of triterpenes may interfere in the antibiofilm activity with minimal or no antibacterial effect. After the screening of 22 compounds at three distinct concentrations, we found antibiofilm activity for eight distinct derivatives without antibiotic effect. In particular, the derivative 2f, with an isopentanoyl ester at position C-3, was an antibiofilm activity against *S. aureus* without any effect upon mammalian cells.

## 1. Introduction

Since the antimicrobial drug class loses effectiveness over time through bacterial resistance, innovation is needed immediately. Bacterial resistance mechanisms are a global problem, and without any effective antibacterial treatment, medical treatment options may be reduced [1]. The biofilm (polymicrobial aggregates such as films) formation is related to many unsuccessful treatments, based on the fact that bacteria biofilm is difficult to treat with only antibiotics [2]. 

Biofilm is formed when a bacterial community attaches to a surface and forms a polymeric matrix of hydrated extracellular polymeric substances [3]. Such body surfaces include, for example, skin, lung and teeth [4] where the biofilm persists even after treatment with several antimicrobial agents [5]. Another example are the biofilms on medical devices, including artificial heart valves, pacemakers and synthetic joints [6]. 

The development in biofilm treatment usually shows that the bacteria in the biofilm mode of growth are less sensitive to antibiotic-mediated killing than the same strain when grown in a free aqueous suspension [7]. Continuing research into new drugs that are active against bacterial biofilm may find a way to decrease the number of infection cases produced by bacteria. 

Such research has essentially shown that there are two different ways to approach the treatment of biofilms: to prevent them from forming or to remove already formed biofilms. Antibiofilm activity has been reported for compounds acting both ways; for example, antimicrobial peptides (peptide AS10), surfactants (surfactin), free fatty acids (oleic acid, *cis*-2-decenoic acid), amino acids (glutamate), indole and its derivatives (indole-3-acetaldehyde), metal chelators (EDTA) and nitric oxide donors (SNP) [8]. Furthermore, another alternative is the terpenes, natural bioactive compounds, several of which have been studied in chemical trials, such as anticancer, anti-inflammatory, antiviral, antimicrobial, antifungal, antihyperglycemic and antiparasitic ones [9,10,11,12,13,14,15].

Belonging to the terpene class, the hydroxyl pentacyclic triterpene ursolic acid (3β-hydroxyurs-12-en-28-oic acid, UA) is an example. Ursolic acid is prevalent in plants as apple (*Malus domestica*) fruit peel. The extraction of UA from apples for juice production represents the biovalorization of industry by-products, which is the principle of green chemistry [15]. Another hydroxyl pentacyclic triterpene is betulinic acid (3β-hydroxy-lup-20(29)-en-28-oic acid, BA), isolated from the stem bark of the Brazilian medicinal plant *Zizyphus joazeiro*, *Ipomea pescaprae* and the leaves of *Syzrgium claviforum* [9]. Moreover, BA was obtained from betulin by chemistry modification at carbon 28 (C-28) [16]. 

**Figure 1:**
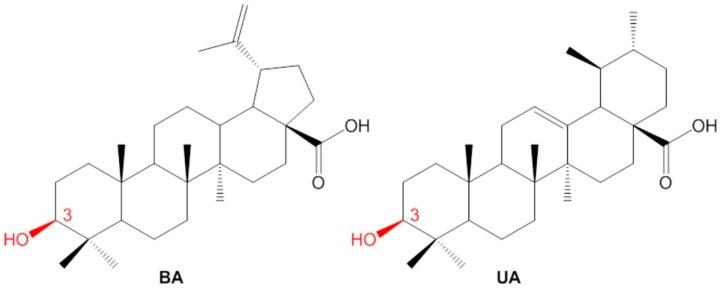
Chemical structure of betulinic acid (BA) and ursolic acid (UA).

**Scheme 1:**
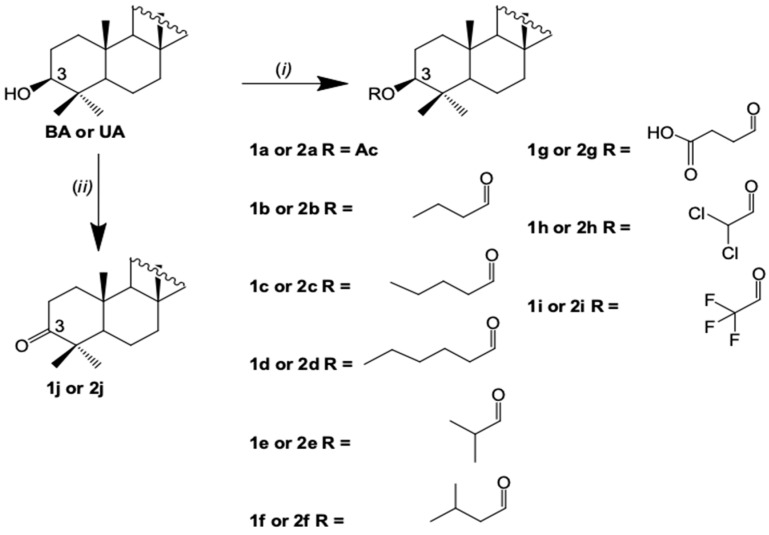
Synthesis of BA (Series 1) and UA (Series 2) derivatives: (*i*) 24h, refluxed, pyridine, succinic anhydride (1g (91%) and 2g (24%)); 1h, room temperature (rt), CH_2_Cl_2_, acetic anhydride (1a (98%) and 2a (100%)), butyric anhydride (1b (25%) and 2b (30%)), pentanoic anhydride (1c (50%) and 2c (56%)), caproic anhydride (1d (47%) and 2d (50%)), isobutyric anhydride (1e (62%) and 2e (45%)); isovaleric anhydride (1f (45%) and 2f (40%)), dichloroacetic anhydride (1h (61%) and 2h (60%)) and bis(trifluoroacetic) anhydride (1i (100%) and 2i (100%)). (ii) 3h, rt, Jone’s reagent/acetone, 1j (85%) and 2j (80%).

Ursolic acid has been described as inhibiting cariogenic microorganisms, by preventing biofilm formation and antibacterial activity on mature biofilms [17]. Previous studies have described UA as having moderate to good antibacterial activity against gram-positive bacteria, *Staphylococcus aureus* and *Enterococcus faecalis* [18]. For this reason, the aim of this present investigation is to evaluate the antibiofilm formation and antibacterial activity of UA and a chemical similar to triterpene BA, (Figure 1) along with semisynthetic derivatives (Scheme 1) with modifications at C-3.

## 2. Materials and Methods 

### 2.1. Isolation of Betulinic Acid and Ursolic Acid

Betulinic acid was isolated from the *Platanus acerifolia* bark (voucher specimen: ICN 171329) and UA was isolated from waste apple (*M. domestica*) peels obtained from a local juice factory [13]. In short, 140 g of *P. acerifolia* bark were extracted with 400 mL of ethanol by reflux. Following this, the ethanol extract was dissolved in H_2_O (100 mL) and extracted successively with dichloromethane and ethyl acetate. After evaporation under vacuum following crystallization using methanol, we obtained BA. In order to obtain UA, the wastes of *M. domestica* were dried and then submitted to extraction using H_2_O following extraction with ethanol by reflux. The extract was evaporated under vacuum, yielding a residue that was chromatographed over silica gel using dichloromethane as an eluent to obtain UA. Both compounds were identified using full spectroscopy data, which was consistent with those previously described [11,13,19]. 

### 2.2. Semisynthesis of the Betulinic Acid and Ursolic Acid Derivatives

Betulinic acid and UA derivatives were synthesized according to the general procedure described in the literature [11,13,19]. In brief, for ester derivatives the commercial anhydride (1.1 mmol, 5 Eq) and 4-(dimethylamino)pyridine (DMAP) (0.22 mmol, 1 Eq) were added to BA or UA (0.22 mmol) in pyridine or CH_2_Cl_2_ (2 mL). Then the reaction was refluxed for 24h (for cyclic anhydrides: succinic anhydride) or processed without refluxing for one hour (for acyclic anhydrides: acetic anhydride, butyric anhydride, pentanoic anhydride, caproic anhydride, isobutyric anhydride, isovaleric anhydride, dichloroacetic anhydride and bis(trifluoroacetic) anhydride). The crude residue was purified in column chromatography by using silica gel 60 (Merck, Billerica, MA, USA) to give the expected pure compounds. For ketone derivatives, the oxidation of the secondary hydroxyl group of BA or UA was performed using the Jones reagent in acetone, for 3h at room temperature. Afterwards, the derivatives were also obtained by purification in silica gel column chromatography. Analytical thin layer chromatography (TLC) was performed on TLC plates silica gel 60 (Merck) and spots were made visible by spraying with anisaldehyde/sulphuric. All compounds obtained are illustrated in Scheme 1. All compounds were identified using full spectroscopy data, which were consistent with those previously described [11,13,19].

### 2.3. Bacteria Strain and Culture Condition

*Staphylococcus epidermidis* ATCC 35984, *S. aureus* ATCC 25904 and *E. faecalis* ATCC 29212 were grown in Mueller Hinton (MH) agar (Oxoid Ltd., Basingstoke, England) overnight, at 37 °C, and a bacterial suspension in 0.9% sterile saline, corresponding to an optical density at 600 nm (OD_600_) of 0.150 (3 × 10^8^ CFU/mL), was used in the assays. The sterile 96-well polystyrene flat-bottom microtiter plates (Corning® Costar® 3599) were purchased from Corning Inc (Corning, NY, USA). 

### 2.4. Antibiofilm and Antibacterial Activity Assays

A planktonic susceptibility testing of *S. epidermidis, S. aureus* and *E. faecalis* was performed by the reference broth microdilution assay according to Clinical and Laboratory Standards Institute (CLSI) guidelines [20]. The growth was evaluated by the difference between the OD600 absorbance measured at the beginning (*t* = 0 h) and at the end (*t* = 24 h) of the incubation time using a 96-well polystyrene flat-bottom microtiter plate and cation-adjusted Mueller-Hinton broth. As a negative control for bacterial growth the derivatives were replaced by 80 µL of water, with this being considered as representing 100% planktonic bacterial growth. Values higher than 100% represent a stimulation of bacterial growth when compared to the control. The minimum inhibitory concentration (MIC) to kill 100% of bacterial cells was determined, and 50 µL of a serial dilution was spread on to MH agar plates. Vancomycin 8 µg/mL (Sigma-Aldrich Co., St. Louis, MO, USA) was used as a positive control for the inhibition of bacterial growth.

The antibiofilm assays were performed with the incubation of the bacterial solution with the compounds in 96-well microtiter plates, as established by Trentin et al. [21]. Firstly, 80 µL of the bacterial suspension, 80 µL of the triterpenes derivatives solution (previously solubilized in DMSO 2% and water) in concentrations of 100, 25 or 5 µM in the wells, and 40 µL of tryptone soya broth (TSB, Oxoid Ltd., Basingstoke, England) were added. After that, the experiment followed the incubation period at 37 °C of 24 h, and after this the content of the wells was removed and washed three times with sterile saline. The remaining attached bacteria were heat-fixed at 60 °C for 1 h. The adherent biofilm layer that was formed was stained with 0.4% crystal violet for 15 min at room temperature. The stain bound to the cells was solubilized with 99.5% DMSO (Sigma-Aldrich Co.) for 30 min, and the absorbance was measured at 570 nm (Spectramax M2e Multimode Microplate Reader, Molecular Devices, San Jose, CA, USA). 

This was used in the antibiofilm formation and antimicrobial assays, negative controls representing 100% of the biofilm formation or 100% of the planktonic cells viability, respectively. In both experiments the positive control used was a commercial antimicrobial vancomycin 8 µg/mL (Sigma–Aldrich Co.), while the vehicle control that was used was a DMSO 2% solution [21,22].

### 2.5. Cytotoxicity Assay

To verify the in vitro cytotoxicity of the compounds, a VERO (Kidney African Green Monkey) cell line ATCC CCL-81 (1 × 10^4^ cell/mL) was incubated using supplemented Dulbecco’s Modified Eagle Medium (DMEM) culture medium (10% *v*/*v* fetal bovine serum, 100 U/mL penicillin and 100 μg/mL streptomycin) for 24 h, at 37 °C in a humidified atmosphere containing 5% CO_2_ in 96-well plates. Following this, the content of the wells was removed and the compound solutions (final concentration in the wells of 100, 25 and 5 µM) and controls, negative (untreated), vehicle (0.1% DMSO) and positive (triton-X 0,1%), were used. At 3h before the desired time, 20 µL of MTT (3-(4,5-dimethylthiazol-2-yl)-2,5-diphenyltetrazolium bromide) solution (5 mg/mL in PBS) was added into each well, and cells were incubated at 37 °C for 3 h. The medium was removed, and 100 µL of DMSO was added into each well. The plate was gently rotated on an orbital shaker for 10 min to completely dissolve the precipitation. The absorbance was detected at 570 nm [23]. 

### 2.6. Statistical Analysis

All experiments were carried out in quintuplicate in three alternated days. The data are presented as a percentage mean ± standard deviation (SD). Differences between groups were evaluated by students’ *t*-test, and a *p* ≤ 0.01 was considered significant.

## 3. Results and Discussion

We report for the first time an activity of hydroxy pentacyclic triterpenes, BA and UA, and their derivatives (Figure 1; Scheme 1) against biofilm formation and bacterial growth. After the screening of 22 triterpene derivatives, we found that the most promising entity was the group isopentanoyl ester at C-3 of the UA scaffold, since it presented a remarkable antibiofilm activity against one of the most important pathogens, *S. aureus*, without antibiotic activity and with no significant cytoxicity activity. The 22 compounds showed different and significant inhibition levels of the biofilm formations. We described the inhibition levels in accordance with Yuyama et al. [24], who consider the range of 70–100% as high, 40–69% as good and 20–39% as moderate inhibition of the biofilm formation.

**Figure 2:**
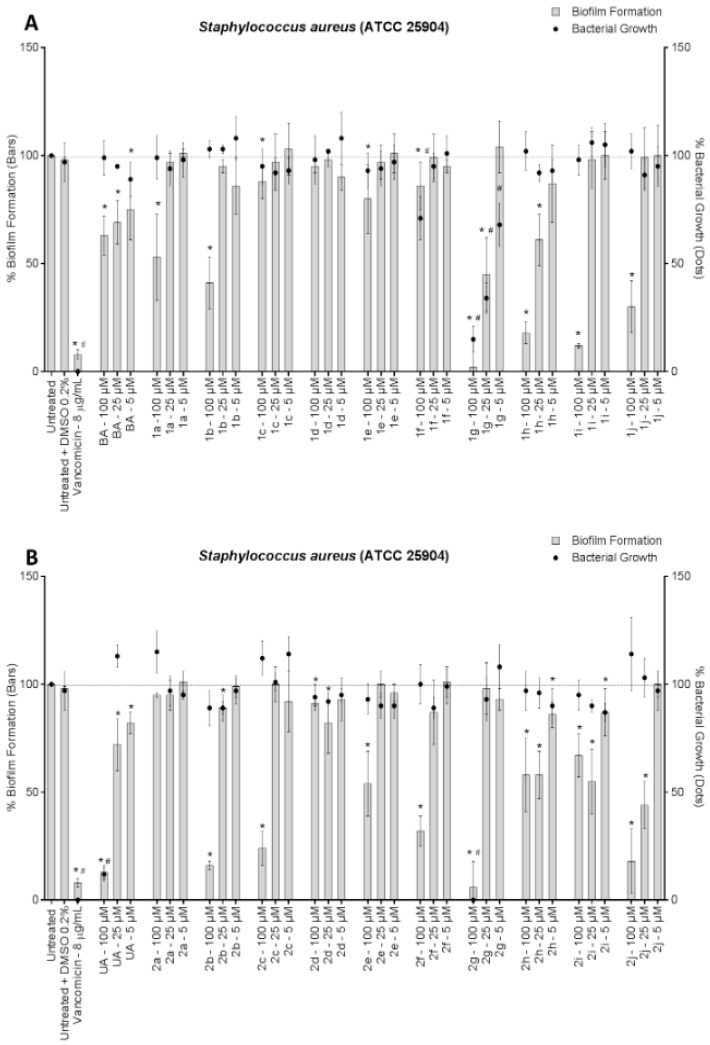
In vitro antibiofilm and antibacterial activity of *Staphylococcus aureus*: (**A**) BA and derivatives; (**B**) UA and derivatives. Results represent mean ± standard deviation of five experiments. * Represents a significant difference in relation to the biofilm formation control (*p* < 0.01). # Represents a significant difference in relation to the planktonic cells’ viability control (*p* < 0.01). BA: betulinic acid; UA: ursolic acid.

Ursolic acid possess no antibiofilm activity (Figure 2), but, at 100 µM against *S. aureus*, UA showed an antibiotic activity. Interestingly, after the specific substitution at position C-3, the antibiotic activity is lost, and an antibiofilm activity is acquired. In this sense, the chemical introduction of isopentanoyl ester 2f (68% of the biofilm inhibition at 100 µM), dichloroacyl ester 2h (42% of the biofilm inhibition at 100 µM) and ketone 2j (82% of the biofilm inhibition at 100 µM) increased the inhibition of the biofilm formation to a good and high range. From this data, chemical modifications and the respective structure–activity relationship (SAR) of UA are potential strategies to improve activity, especially against biofilm formation.

Additionally, investigations of UA in combination with antibiotics have been conducted as a good alternative for the treatment of bacteria that is medically relevant. For example, Wojnicz et al. [25] described the combination of UA and ciprofloxacin (used for the treatment of recurring urinary tract infections caused by *Escherichia coli*) and showed an enhanced antibiofilm effectiveness against *E. coli*, which may be related to the acidic character of UA. Hence, obtaining UA derivatives with a low toxicity and strong biofilm inhibition to be used in combination with antibiotics may become an option for the treatment of bacterium infections.

**Figure 3:**
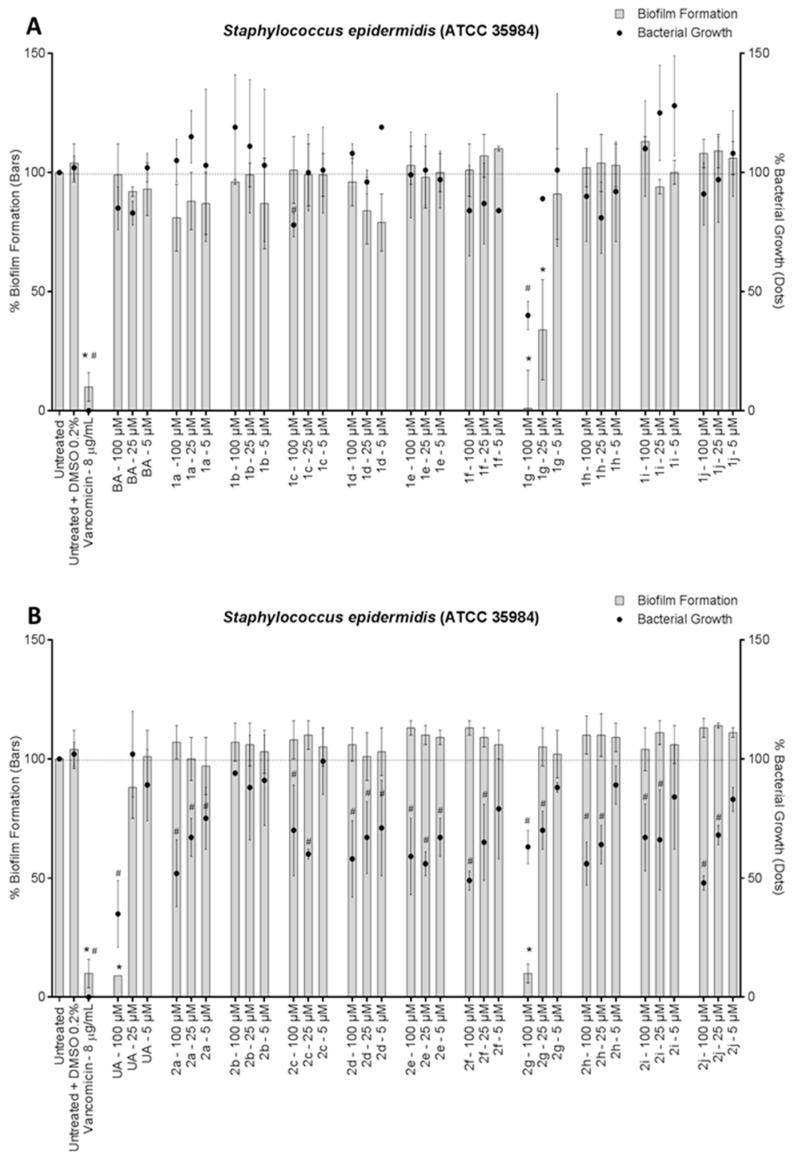
In vitro antibiofilm and antibacterial activity of *Staphylococcus epidermidis*: (**A**) BA and derivatives; (**B**) UA and derivatives. Results represent mean ± standard deviation of five experiments. * Represents significant difference in relation to biofilm formation control (*p* < 0.01). # Represents significant difference in relation to planktonic cells viability control (*p* < 0.01).

**Figure 4:**
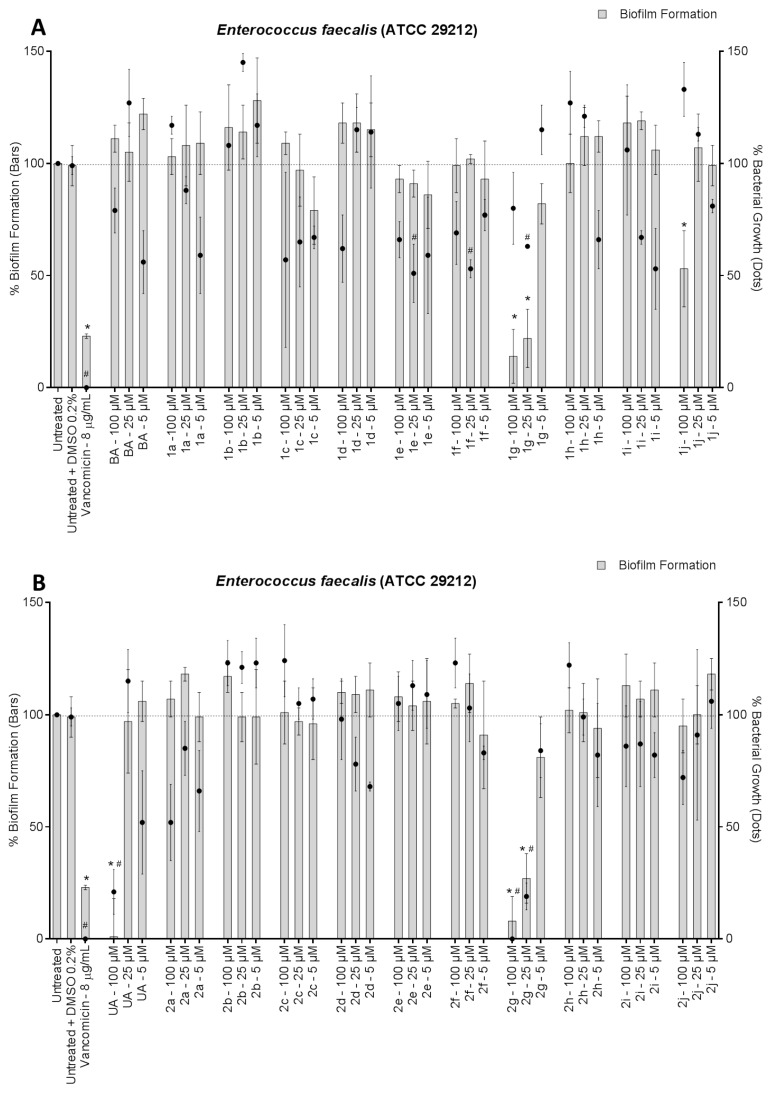
In vitro antibiofilm and antibacterial activity of *Enterococcus faecalis*: (**A**) BA and derivatives; (**B**) UA and derivatives. The results represent the mean ± standard deviation of five experiments. * Represents a significant difference in relation to the biofilm formation control (*p* < 0.01). # Represents a significant difference in relation to the planktonic cells’ viability control (*p* < 0.01).

On the other hand, the BA possess an antibiofilm activity (without any antibiotic activity) displaying a moderate inhibition of the biofilm formation in *S. aureus* of 37% at 100 µM, 31% at 25 µM and 25% at 5 µM, with a significant difference in relation to the biofilm formation control (*p* < 0.01) (Figure 2). However, BA did not demonstrate an antibiofilm activity against other gram-positive bacteria tested (*E. faecalis* and *S. epidermidis*) (Figure 3 and Figure 4). After the substitution at the C-3 position, the antibiofilm activity increased for five derivatives, namely 1a (acetyl ester), 1b (propyl ester), 1h (dichloroacyl ester), 1i (trifluoracyl ester) and 1j (ketone). A high percentage of the biofilm inhibition was observed, particularly against *S. aureus*, but *E. faecalis* and *S. epidermidis* were less sensitive to these derivatives. The derivatives 1a (47% at 100 µM) and 1b (59% at 100 µM) showed a good inhibition of the biofilm formation in *S. aureus*, whereas 1h (82% at 100 µM), 1i (88% at 100 µM), and 1j (70% at 100 µM) showed a high inhibition of the biofilm formation in *S. aureus* (Figure 2). Regarding *S. epidermidis*, only 1g (66% at 25 µM) showed a good inhibition of the biofilm formation (Figure 3), whereas 1j (47% at 100 µM) against *E. faecalis* is included in the same range (Figure 4). Following this, these derivatives were more active than their precursor BA, and 1i is the most active, showing that the presence of electronegative groups at C-3 may mainly enhance the antibiofilm activity.

Although the triterpenes had previously been proposed as being relatively non-toxic, [26,27] the effect of BA and UA on two eukaryotic cell lines (HaCaT and MRC-5) had been examined; they showed a higher BA cellular impact than UA [18]. From the conclusion that the use of such pure compounds is not harmless, a chemical modification in order to obtain safe compounds might be an alternative. Alongside this information and the fact that biofilms are very difficult to eradicate, the aim of this present investigation is to find not only a new and more efficient compound against biofilm formation with minimal or no antibiotic effect, but also compounds with low cytotoxicity. Our data showed that the derivatives synthesized increased the VERO cell viability compared to precursors BA and UA, except for 1h (100 µM), 1i (100 µM) and 2i (100 and 25 µM). The UA derivatives, 2f and 2h, displayed better results without any effect upon mammalian cells at all the tested concentrations (100, 25 and 5 µM) (Table 1). In a previous study, we reported that 1b, 2b and 1e derivatives were non-cytotoxic at tested concentrations >100 µM against another mammalian cells, HEK293T (human embryonic kidney) cells [13]. Following this, the chemical modification at C-3 is also justified by the obtaining of safer compounds. Follow-up studies with non-cytotoxic compounds are important, as adverse effects have been described in antibiotic uses [28]. 

The mechanism of action was not elucidated, which will be our next step, along with a further investigation of the combination of triterpenes derivatives with antibiotics, which may become an interesting alternative for the treatment of gram-positive bacterial infections in the future. Considering all the observed activities, we concluded that the most promising compound is the 2f, with an isopentanoyl ester at position C-3. The results bring 2f a step closer to the ideal compound for evaluating in combination with antibiotics and in in vivo experiments, since this derivative is not cytotoxic like its precursor and other derivatives, non-anti-bacterially active and effective against biofilm formation. 

An additional information showed by our derivatives was the antibiotic activity, which deserves thorough future investigation, since it is not the focus of this study. Ursolic acid showed an antibiotic activity against *S. aureus*, *S. epidermidis* and *E. faecalis* at 100 µM, and BA was non-active against these bacteria (Figure 2, Figure 3 and Figure 4). Betulinic acid and UA are chemically similar, but the antibacterial and antibiofilm activity spectrum is different. Overall, UA derivatives displayed a better antibiotic activity than BA derivatives, especially against *S. epidermidis* (Figure 3). For UA derivatives, succinyl ester 2g reduced the planktonic cells viability of all gram-positive tested strains (*S. epidermidis* (100 and 25 µM), *S. aureus* (100 µM) and *E. faecalis* (100 and 25 µM)). For BA series, succinyl ester 1g reduced the planktonic cells viability of *S. epidermidis* (100 µM), *S. aureus* (100, 25 and 5 µM) and *E. faecalis* (25 µM). Focusing on the SAR, it is important to note the role of the derivative possessing the succinic acid group at C-3: it is the most active in both BA and UA. In view of these findings, modifications at the C-3 position of BA and UA showed the influence of the presence of polar or hydrophilic groups (carboxylic acids), and their ability to impair planktonic cells viability more than side-chain lengths can. 

## 4. Conclusions

In brief, BA, UA and twenty triterpene derivatives have been synthetized and evaluated in vitro for the first time against the biofilm of the Gram-positive bacteria strains *S. epidermidis*, *S. aureus* and *E. faecalis*. For these species, several studies have shown their pathogenic efficacy, while new medicine in this field are urgently required. Importantly, we have shown that a substitution of a triterpene at position C-3 directly influences the raise of antibiofilm activity; this includes the fact that after C-3 substitutions the BA with antibiotic activity became antibiofilm derivatives. The ideal antibiofilm compound must be absent of antibiotic activity. In our data, eight compounds seem to be ideal; however, 2f is worthy for further studying, since any effect upon mammalian cells at all tested concentrations (5, 25 and 100 µM) was observed. Here, in vitro screening already provides an important guide to the development of new bioactive compounds derived from natural sources as a future potential. 

## Figures and Tables

**Table 1:** Citotoxity activity of BA and UA and derivatives against VERO cells.

**VERO Cells Viability (%) of Compounds**									
	**100 µM**	**25 µM**		**5 µM**		**100 µM**		**25 µM**	**5 µM**
**BA**	25 ± 3 *	30 ± 3 *		40 ± 6*	**UA**	13 ± 12 *		32 ± 7 *	37 ± 4 *
**1a**	31 ± 8 *	35 ± 11 *		47 ± 12 *	**2a**	55 ± 30 *		74 ± 14	73 ± 10 *
**1b**	30 ± 7 *	34 ± 11 *		46 ± 12 *	**2b**	60 ± 15 *		76 ± 15	90 ± 13
**1c**	27 ± 10 *	33 ± 16 *		50 ± 14 *	**2c**	64 ± 20 *		92 ± 6	68 ± 20 *
**1d**	41 ± 20 *	56 ± 17 *		66 ± 8 *	**2d**	55 ± 4 *		67 ± 7 *	65 ± 11 *
**1e**	29 ± 11 *	64 ± 13 *		63 ± 21 *	**2e**	52 ± 18 *		94 ± 18	100 ± 16
**1f**	58 ± 9 *	60 ± 5 *		63 ± 14*	**2f**	72 ± 26		105 ± 10	113 ± 32
**1g**	67 ± 8*	82 ± 17		56 ± 4 *	**2g**	61 ± 15 *		46 ± 6 *	52 ± 3 *
**1h**	23 ± 5 *	35 ± 13 *		92 ± 6	**2h**	81 ± 11		81 ± 35	86 ± 21
**1i**	10 ± 4 *	72 ± 10		61 ± 7 *	**2i**	4 ± 0 *		28 ± 5 *	69 ± 7 *
**1j**	36 ± 18 *	57 ± 10 *		52 ± 3 *	**2j**	45 ± 3 *		87 ± 8	64 ± 16 *
**VERO Cells Viability (%) of Controls**									
**Negative (Untreated)**			**Vehicle (DMSO 0.1%)**				**Positive Control (Triton-X 0.1%)**		
100 ± 21			91 ± 20				3 ± 0 *		

The results represent the mean ± standard deviation of five experiments. * Represents a significant difference in relation to the control (*p* < 0.01). VERO cells: kidney African green monkey cell.
